# An NK cell line (haNK) expressing high levels of granzyme and engineered to express the high affinity CD16 allele

**DOI:** 10.18632/oncotarget.13411

**Published:** 2016-11-17

**Authors:** Caroline Jochems, James W. Hodge, Massimo Fantini, Rika Fujii, Y. Morillon Maurice, John W. Greiner, Michelle R. Padget, Sarah R. Tritsch, Kwong Yok Tsang, Kerry S. Campbell, Hans Klingemann, Laurent Boissel, Shahrooz Rabizadeh, Patrick Soon-Shiong, Jeffrey Schlom

**Affiliations:** ^1^ Laboratory of Tumor Immunology and Biology, Center for Cancer Research, National Cancer Institute, Bethesda, MD, USA; ^2^ Immune Cell Development and Host Defense Program, Institute for Cancer Research, Fox Chase Cancer Center, Philadelphia, PA, USA; ^3^ NantKwest, Inc., Culver City, CA, USA; ^4^ NantCell, LLC, Culver City, CA, USA

**Keywords:** ADCC, NK lysis, immunotherapy, high affinity CD16, cetuximab

## Abstract

Natural killer (NK) cells are known to play a role in mediating innate immunity, in enhancing adaptive immune responses, and have been implicated in mediating anti-tumor responses via antibody-dependent cell-mediated cytotoxicity (ADCC) by reactivity of CD16 with the Fc region of human IgG1 antibodies. The NK-92 cell line, derived from a lymphoma patient, has previously been well characterized and adoptive transfer of irradiated NK-92 cells has demonstrated safety and shown preliminary evidence of clinical benefit in cancer patients. The NK-92 cell line, devoid of CD16, has now been engineered to express the high affinity (ha) CD16 V158 FcγRIIIa receptor, as well as engineered to express IL-2; IL-2 has been shown to replenish the granular stock of NK cells, leading to enhanced perforin- and granzyme-mediated lysis of tumor cells. The studies reported here show high levels of granzyme in haNK cells, and demonstrate the effects of irradiation of haNK cells on multiple phenotypic markers, viability, IL-2 production, and lysis of a spectrum of human tumor cells. Studies also compare endogenous irradiated haNK lysis of tumor cells with that of irradiated haNK-mediated ADCC using cetuximab, trastuzumab and pertuzumab monoclonal antibodies. These studies thus provide the rationale for the potential use of irradiated haNK cells in adoptive transfer studies for a range of human tumor types. Moreover, since only approximately 10% of humans are homozygous for the high affinity V CD16 allele, these studies also provide the rationale for the use of irradiated haNK cells in combination with IgG1 anti-tumor monoclonal antibodies.

## INTRODUCTION

Natural killer (NK) cells are essential in mediating innate immune responses and in the enhancement of the adaptive immune response [1–3]. In addition to these mechanisms, NK cells can play a role in anti-tumor immunity alone, or in combination with select antibodies via antibody-dependent cell-mediated cytotoxicity (ADCC). Several NK cell lines have been derived from patients with a range of different leukemias and lymphomas [4]. One such NK line, designated NK-92, derived from a patient with non-Hodgkin's lymphoma, has been previously well characterized [5–7]. NK-92 cells grow well in culture and are dependent on exogenous IL-2 for propagation. They also lack or have extremely low levels of the inhibitor killer immunoglobulin (Ig)-like receptors (KIR), which allows for lysis of tumor cells expressing major histocompatibility complex (MHC) molecules. Preclinical studies have shown that NK-92 cells do not form tumors when transplanted into severe combined immunodeficiency (SCID) or athymic mice.

Several clinical studies employing repeated infusions of irradiated NK-92 cells have been completed. Up to 10 billion cells/m^2^ have been infused with no severe side effects observed [8, 9]. Clinical responses have been observed in patients with melanoma, lung cancer, Merkel cell carcinoma, lymphoma, and kidney cancer [4, 8, 9]. Despite the allogeneic nature of NK-92 cells, the formation of anti-human leukocyte antigen (HLA) antibodies was seen in less than half of the patients. Moreover, the pharmacodynamics of NK-92 clearance did not differ upon repeated doses. NK-92 cells, however, do not express the CD16 Fc receptor, which is necessary for NK-mediated ADCC lysis of tumor cells employing monoclonal antibodies (MAbs) of the immunoglobulin G1 (IgG1) isotype. NK cells from healthy donors can express the endogenous alleles for the CD16 valine (V) high affinity Fc receptor FcγRIIIa (158V) only (V/V genotype), for the lower affinity phenylalanine (F) allele only (F/F genotype), or express both (V/F genotype). *In vitro* studies of donor NK cells, using tumor cells as targets, have generally shown higher levels of tumor cell cytotoxicity using NK cells from patients with the V/V genotype vs. NK cells from V/F or F/F genotypes.

Prior clinical studies [10–13] employing the IgG1 isotype MAbs cetuximab (Erbitux), trastuzumab (Herceptin), or rituximab (Rituxan) have shown that colorectal cancer, breast cancer, and lymphoma patients, respectively, whose NK cells express CD16 V allele only (V/V), have improved overall survival compared to patients with NK cells expressing the V/F or F/F alleles. While there is no way to prove that the enhanced clinical benefit in the use of these monoclonals is, in part, contributed by the ADCC mechanism, the data remain somewhat compelling. One issue, however, is that only approximately 10% of the population is homozygous for the high affinity V allele [14].

NK-92 cells have now been engineered to express the CD16 high affinity FcγRIIIa (158V) receptor [15]. This modified NK-92 cell line has been designated haNK (high affinity NK). haNK cells have also been engineered to endogenously express IL-2 to circumvent the need for culture with exogenous IL-2. NK cells have previously been shown [16, 17] to be “serial killers,” in that a single NK cell can lyse multiple tumor cells. These studies have also shown [16, 17] that IL-2 can replenish the granular stock of NK cells leading to enhanced perforin- and granzyme-mediated lysis of “exhausted” NK cells.

The engineered CD16 high affinity Fc receptor and endogenous IL-2 in haNK cells may enhance the potential clinical utility of these cells. However, since the parent NK-92 cells were originally derived from a lymphoma patient, haNK cells will require lethal irradiation prior to any clinical use. This study is designed to describe the phenotype of haNK cells, and if changes in phenotype exist post-irradiation. Also described are the characteristics of the endogenous lytic activity *in vitro* of irradiated haNK cells toward a range of human tumor cells, and the use of irradiated haNK cells in ADCC-mediated lysis of tumor cells employing three widely used anti-tumor MAbs.

## RESULTS

**Figure 1:**
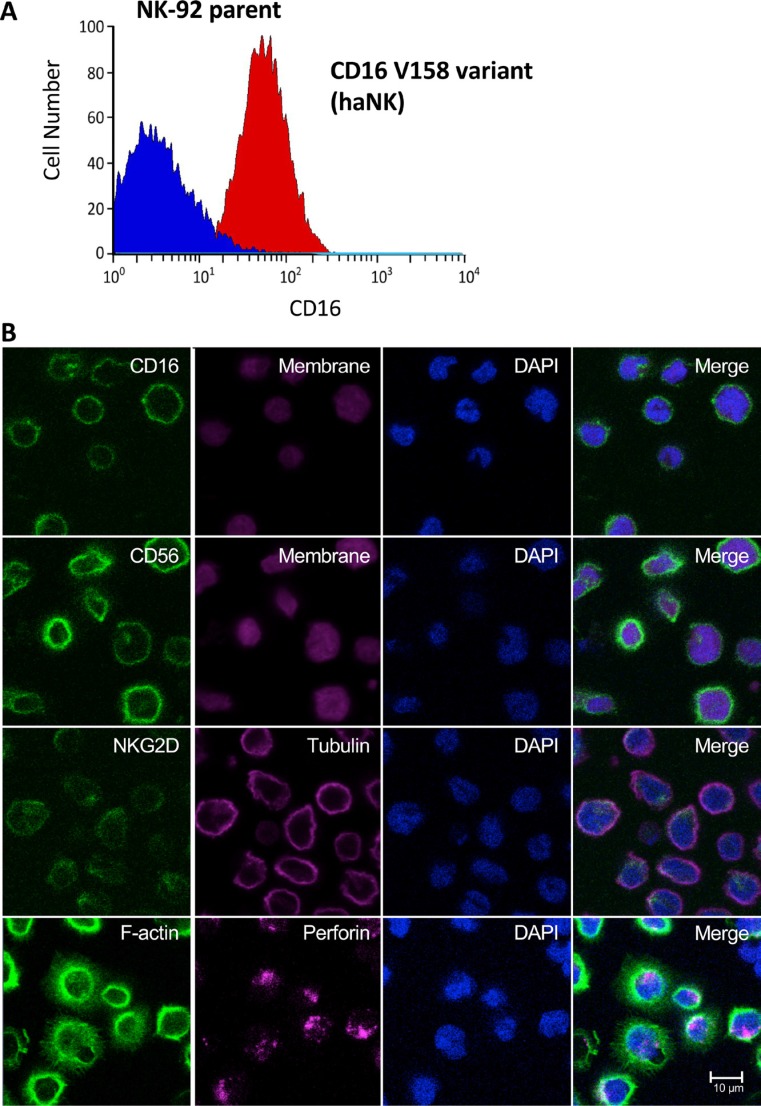
Analyses of CD16 high affinity variant (V158) in haNK cells (**A**) The NK-92 parent cell line was modified to express a high affinity CD16 variant. (**B**) Immunofluorescence imaging of haNK cells. haNK cells were stained for expression of common NK markers as described in Materials and Methods. The expression of CD16 (green), CD56 (green), NKG2D (green), F-actin (green), CellMask plasma membrane stain (magenta), tubulin (magenta), perforin (magenta), and DAPI nuclear stain (blue) were visualized by confocal microscopy. Scale bar = 10 μm.

As described in the Methods section, NK-92 cells have been engineered to endogenously express IL-2. This enables haNK cells to be propagated in culture without the need to provide exogenous IL-2. As detailed previously [16], the addition of exogenous IL-2 also has the ability to replenish the granular stock of NK cells, leading to an increase in granzyme B content. As shown previously [18], NK-92 cells have considerably higher levels of endogenous granzyme when compared to NK cells or IL-22-activated NK cells. haNK cells have also been engineered to express the high affinity CD16 Fc receptor FcγRIIIa (158V). As shown in Figure 1A, haNK cells express high levels of the CD16 158V variant, while the parent NK-92 cells do not. Figure 1B shows confocal images of haNK cell expression of CD16, CD56, NKG2D, and perforin.

**Figure 2:**
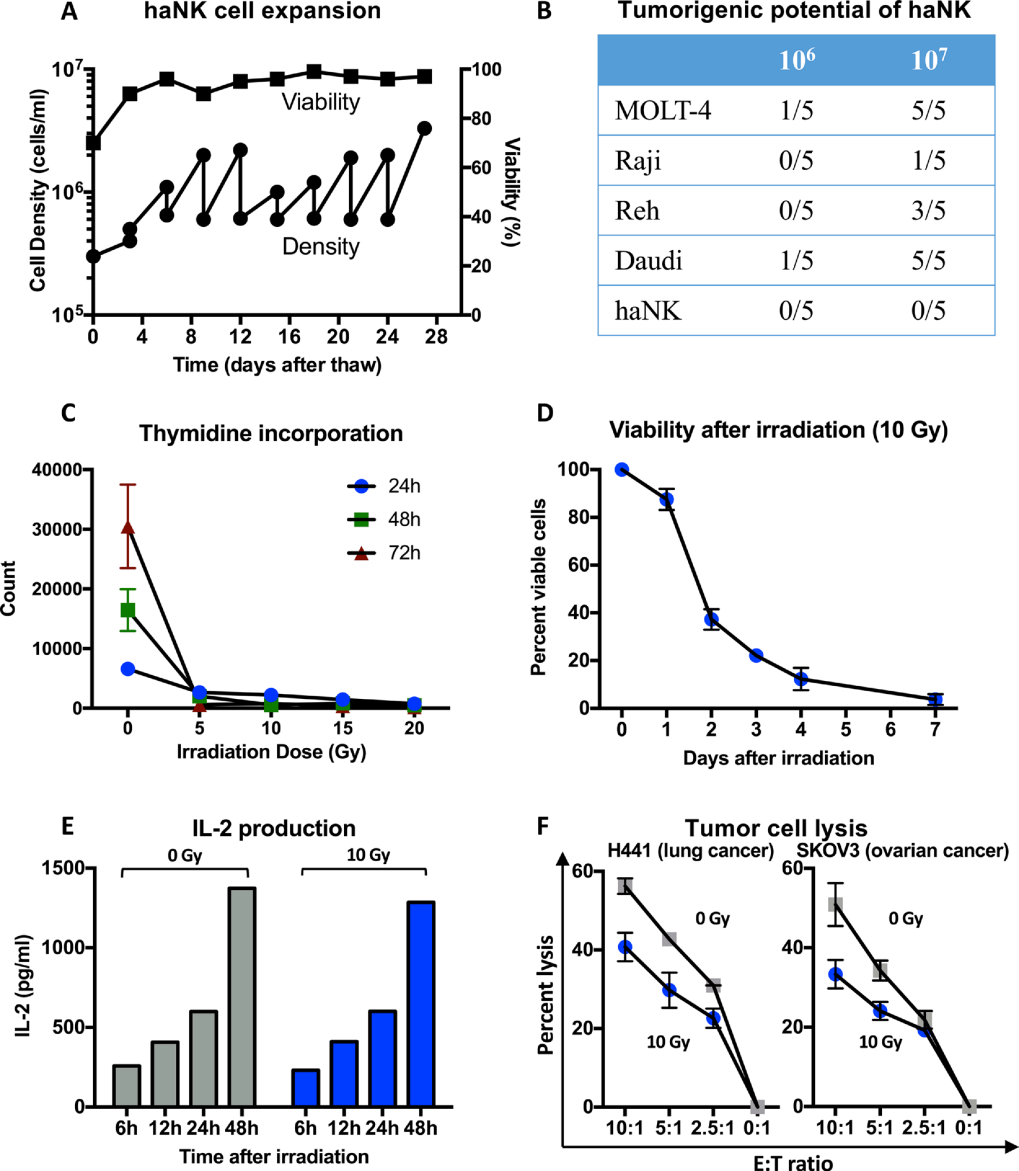
Characteristics of haNK cells before and after irradiation (**A**) haNK cell expansion (subcultured every 3 days) with cell density (cells/ml) and viability (%). haNK cells were cultured in X-VIVO-10 medium without phenol red, and followed for 28 days. (**B**) Evaluation of the tumorigenic potential of non-irradiated haNK cells. Male athymic nude mice were injected s.c. with 10^6^ or 10^7^ of different tumor cells (see Methods), and monitored daily for the presence of tumors at injection sites. At study conclusion (day 63) the number of resultant viable tumors (≥ 50 mm^3^) was quantified for each tumor cell line and cell number injected. (**C**) ^3^H-thymidine incorporation was measured to evaluate proliferation of haNK cells irradiated at different doses. Irradiation at ≥ 5 Gy was adequate to render haNK cells replication incompetent. (**D**) haNK cell viability was evaluated daily after irradiation (10 Gy) using AO/PI staining. (**E**) Evaluation of haNK cell cumulative cytokine secretion after irradiation (10 Gy). Results are in pg/ml for 5 × 10^5^ haNK cells/ml. (**F**) haNK cell lysis of the human carcinoma cell lines H441 (lung) and SKOV3 (ovarian) before irradiation (gray squares), and 24 h after irradiation (10 Gy, blue circles) using an 18-h ^111^In release assay.

As seen in Figure 2A, haNK cells can reproducibly be passaged in culture while maintaining virtually 100% viability. Since the parental NK-92 cell line was derived from a lymphoma patient, viable haNK cells were analyzed for tumorigenicity by inoculation into athymic mice at both 10^6^ and 10^7^ cells/mouse and were monitored daily for 63 days for tumor formation. The MOLT-4, Raji, Reh, and Daudi leukemia/lymphoma cell lines were also inoculated similarly into athymic mice. As seen in Figure 2B, while tumors arose in mice transplanted with other tumor cell lines, no tumors arose in mice transplanted with haNK cells. To ensure safety in any potential clinical use, haNK cells were irradiated at doses of 5-20 Gy and analyzed for subsequent proliferation via ^3^H-thymidine incorporation. As seen in Figure 2C, doses of 5 Gy and above were sufficient to eliminate proliferation. The use of 10 Gy was thus chosen as the dose of radiation for subsequent studies. When analyzed daily by AO/PI staining, the percentage of viable haNK cells was reduced to zero at 7 days post-irradiation (Figure 2D).

haNK cells were also analyzed for endogenous IL-2 production post-10 Gy irradiation. As seen in Figure 2E, irradiated haNK cells continued to produce levels of IL-2 comparable to non-irradiated haNK cells, and both non-irradiated and irradiated haNK cells continued to produce IL-2 for at least 48 h. In addition, irradiated haNK cells continued to produce IFN-γ and IL-8 for at least 48 h. Studies were then conducted to determine if irradiated haNK cells maintained cytotoxicity. As seen in Figure 2F, non-irradiated haNK cells efficiently lysed human H441 lung carcinoma cells and human SKOV3 ovarian carcinoma cells, respectively, at various effector to target (E:T) ratios (gray squares). Irradiated haNK cells (blue circles) maintained cytotoxic ability for both of these tumor cell lines, but at approximately 20% lower cytotoxic levels at the various E:T ratios compared to intact haNK cells.

**Figure 3:**
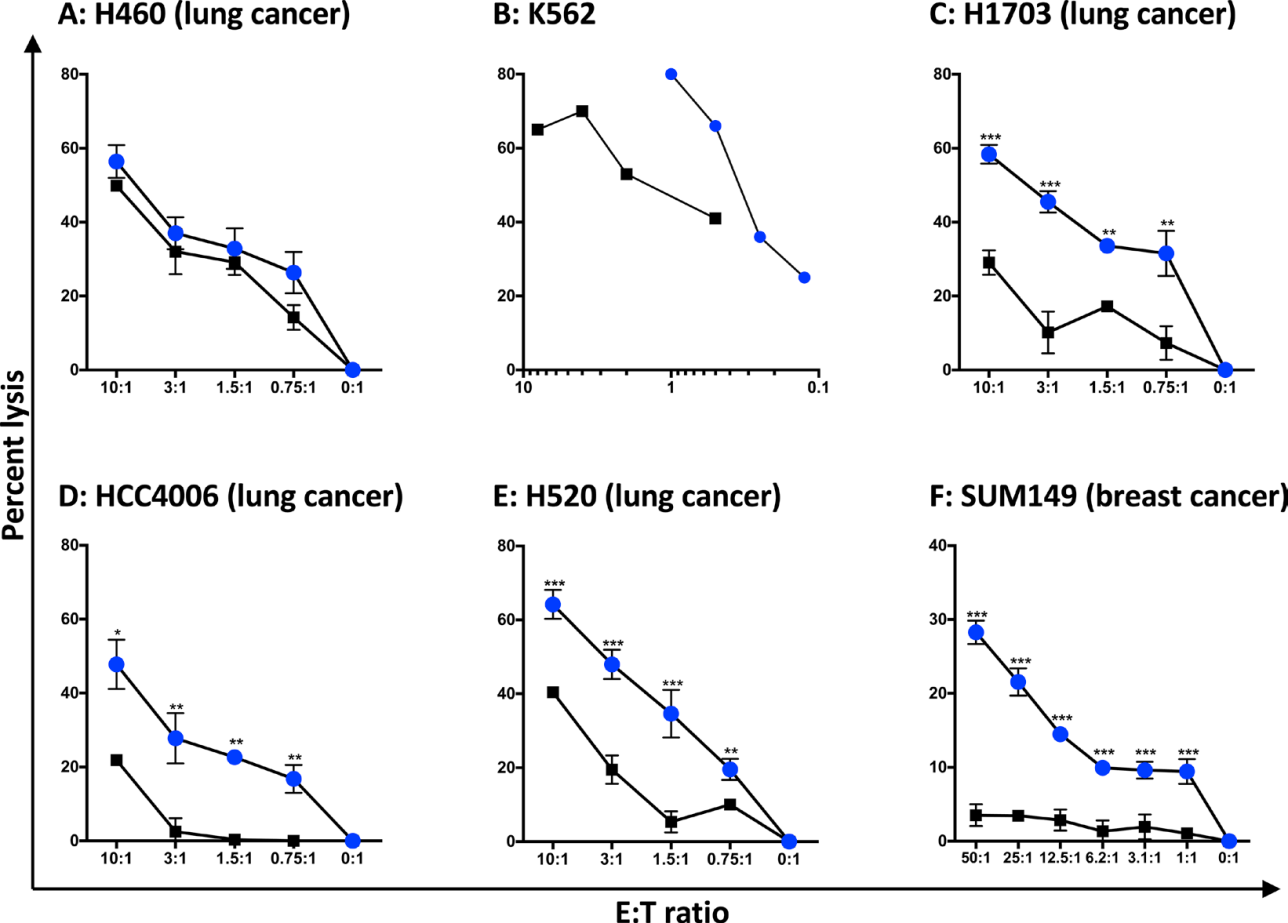
Comparison of tumor cell lysis by haNK cells 1 h vs. 24 h post-irradiation (**A**–**F**) haNK cells were either irradiated (10 Gy) and assayed immediately (squares) or after 24 h in culture (circles). The 18-h haNK lysis assay was performed with different tumor cell targets at the E:T ratios shown. K562 was analyzed by flow cytometry. The human lung cancer cell lines (H460, H1703, HCC4006, and H520) were analyzed by CeligoS, and the human breast cancer cell line SUM149 was analyzed by ^111^In-release. Results shown are the means (SD) of triplicate measurements from 1 of 3 comparable repeat experiments. *T*-tests were employed to compare lysis at 0 h and 24 h post-irradiation at all E:T ratios. ***P < 0.001, **P < 0.01, *P < 0.05.

Since the effect of irradiation on stress of NK cells is unknown, the lytic potential of haNK cells immediately (approximately 1 h) after irradiation vs. 24 h post-irradiation was examined. While no difference was observed in lytic ability of haNK cells at 1 h vs. 24 h post-irradiation using H460 human lung carcinoma cells as targets (Figure 3A), there were substantial differences using four other human carcinoma cell lines as well as K562 cells as targets, with haNK cells 24 h post-irradiation showing greater lytic activity at all E:T ratios compared to immediately after radiation (Figure 3B–3F). Subsequent studies with haNK cells were thus conducted approximately 24 h post-irradiation.

**Figure 4:**
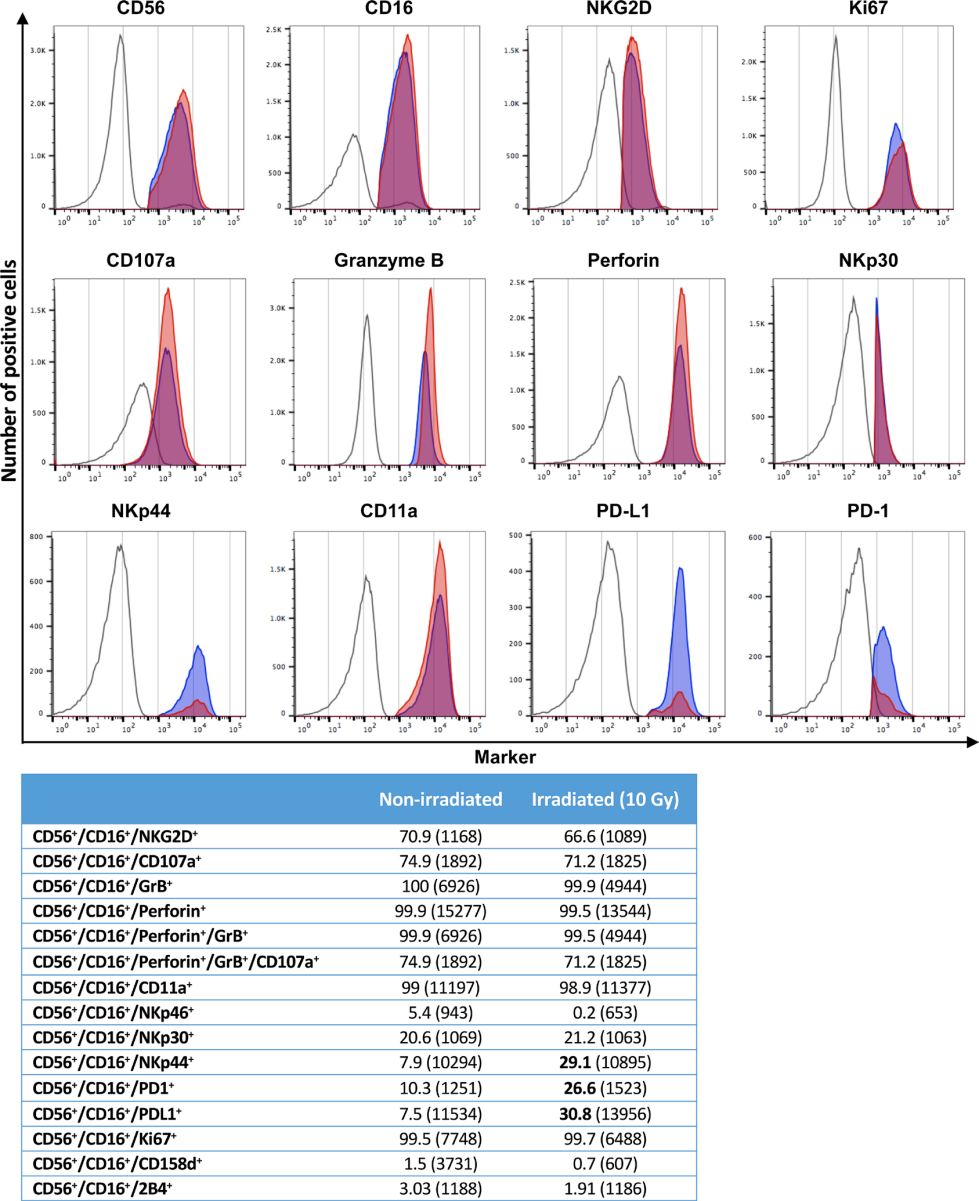
Phenotyping of haNK cells with and without irradiation haNK cells were stained for flow cytometry either non-irradiated, or irradiated (10 Gy) and rested for 24 h before staining. Figures show the expression of different markers for FMO controls (open), non-irradiated haNK cells (red), and irradiated haNK cells (blue). Table: Values shown are percent of CD56^+^CD16^+^ haNK cells (MFI). Bolded figures denote a significant increase after irradiation.

We then interrogated the effect of 10 Gy on the haNK cell phenotype 24 h post-irradiation. As seen in the histograms of Figure 4, the major markers of NK cells did not change post-irradiation, including CD56, CD16, NKG2D; levels of granzyme B, perforin, CD107a, as well as Ki67 and NKp30 also did not change. There was an increase post-irradiation, however, in the levels of NKp44, programmed cell death protein-1 ligand (PD-L1) and programmed cell death protein-1 (PD-1). The potential significance of these changes post-irradiation will be discussed below. The quantification in terms of the percentage of cells positive and mean fluorescence intensity (MFI; in parentheses) of the various markers analyzed via histograms is shown in the lower panel of Figure 4, along with those of several other phenotypic markers.

**Figure 5:**
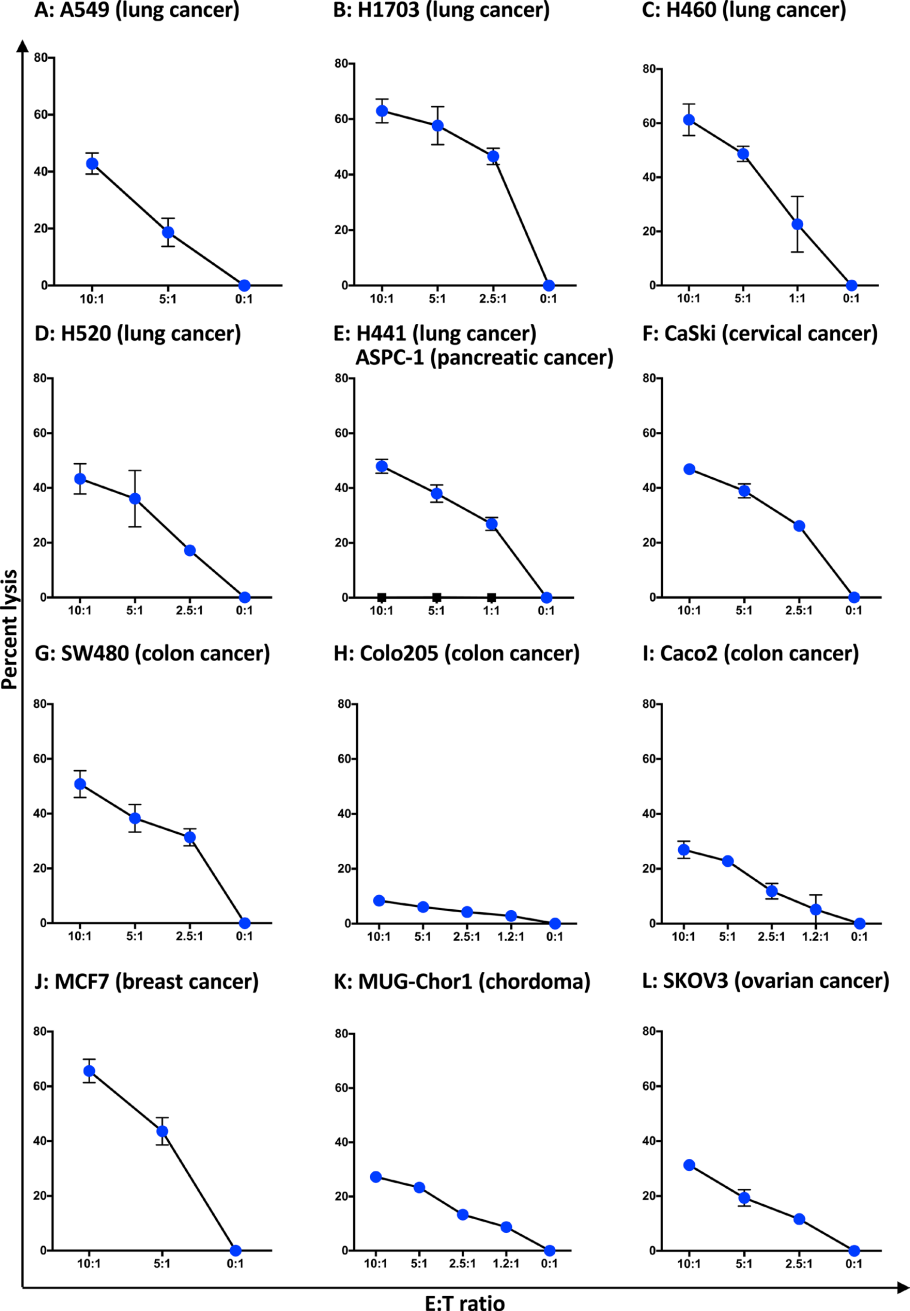
haNK lysis of human tumor cell lines haNK cells were irradiated (10 Gy) and kept in culture for 24 h prior to an 18-h lysis assay at different E:T ratios. Four lung cancer cell lines A549, H1703, H460, and H520 were analyzed by CeligoS (**A**–**D**). In a separate series of studies, human tumor cell line lysis was analyzed by ^111^In-release (**E**–**L**). Results shown are the means (SD) of triplicate measurements from one of at least three comparable repeat experiments.

The ability of irradiated haNK cells to lyse 13 human tumor cell lines was analyzed, including lung (*n* = 5), colon (*n* = 3), breast, cervical, ovarian and pancreatic carcinoma lines, and a chordoma cell line. As seen in Figure 5A–5L, various degrees of lysis of the 12 human tumor cell lines was observed; however, no lysis of the human pancreatic cell line ASPC-1 could be achieved at any E:T ratio (Figure 5E, squares).

The potential reason(s) for differences in the percentage of lysis seen with irradiated haNK cells using different tumor cell lines was investigated. The percentage of lysis by haNK cells at a 10:1 ratio of eight carcinoma cell lines was evaluated relative to the tumor cell lines’ expression of MHC class I chain-related protein A (MICA), PD-L1, and MHC class I, both in terms of percentage of cells expressing each marker and MFI. In Table 1, cells are ranked by percentage of haNK cell lysis. While there are trends in tumor cell lysis with higher levels of MICA and PD-L1 expression, there are clear outliers. The ASPC-1 line, which could not be lysed by haNK cells in numerous experiments, did show the lowest levels of both MICA and PD-L1 expression.

**Table 1:** Tumor cell lysis by irradiated haNK cells, and tumor cell expression of MICA, PD-L1, and MHC class I

Tumor cell line	Tumor type	% MICA (MFI)	% PD-L1 (MFI)	% MHC I (MFI)	% haNK lysis (SD)
**CaSki**	Cervical	99.3 (10,229)	99.9 (20,015)	ND	46.5 (4.9)
**HN30**	Head & neck	2.3 (12,995)	99.8 (21,328)	98.7 (10,963)	45.3 (3.9)
**HTB1**	Bladder	73.1 (4,755)	98.4 (17,225)	99.5 (10,154)	37.6 (2.5)
**HN4**	Head & neck	25.2 (8,842)	99.7 (10,005)	99.5 (9,232)	34.0 (5.3)
**HN12**	Head & neck	36.8 (27,815)	100 (5,177)	96.5 (9,603)	31.0 (2.0)
**Colo205**	Colorectal	7.25 (2,001)	95.7 (1,765)	98.6 (745)	23.4 (2.1)
**SW403**	Colorectal	2.3 (10,176)	51.3 (5,176)	ND	8.6 (2.7)
**ASPC-1**	Pancreatic	0.3 (8,592)	24.0 (3,101)	98.8 (3,060)	0 (1.5)

Tumor cell lines were phenotyped by flow cytometry and used as targets in 18 h ^111^In-release haNK lysis assays using irradiated (10 Gy) haNK as effectors. The effector to target ratio was 10:1. MICA, PD-L1 and MHC class I expression are shown as % positive cells (MFI). ND = not done.

**Figure 6:**
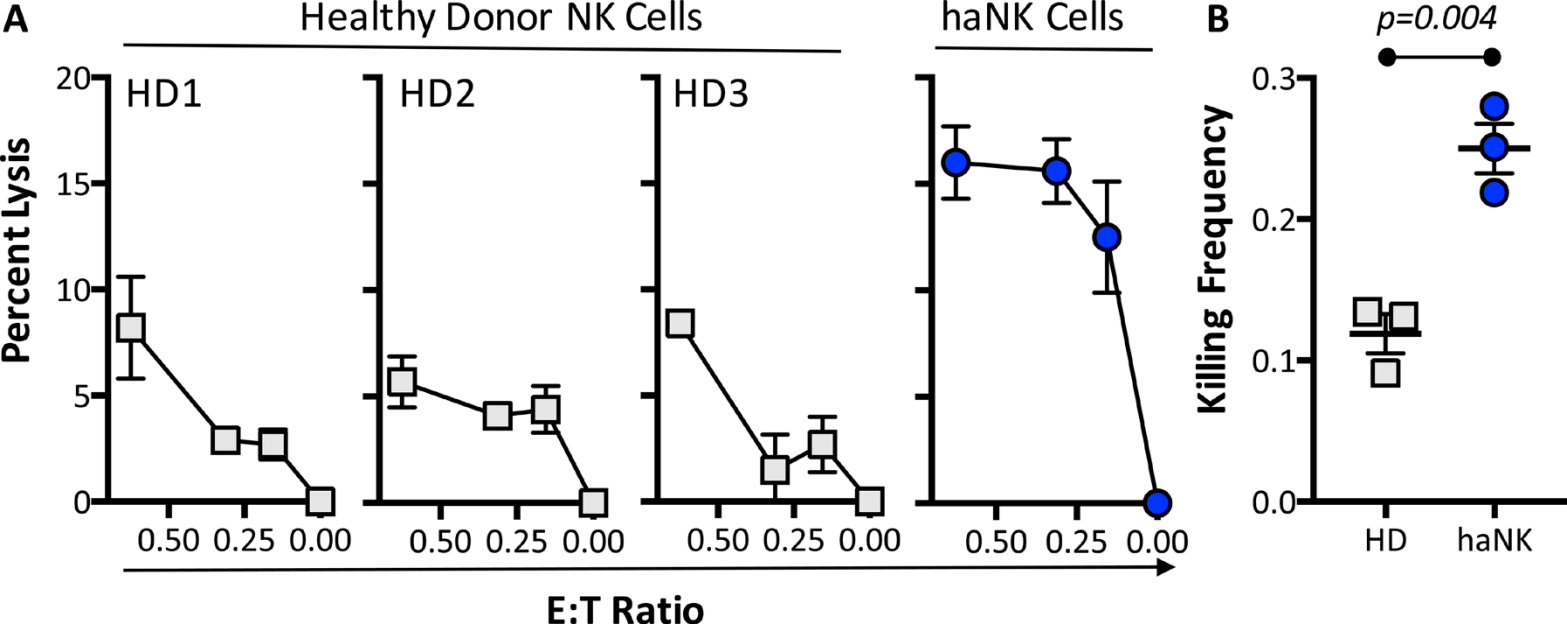
haNK cells have a higher killing frequency than healthy donor NK cells (**A**) Primary human NK cells purified from three independent healthy donors (HD) or haNK (10 Gy irradiated) cells were co-incubated with ^111^In-labeled MDA-MB-231 (breast cancer) target cells at low E:T ratios for 18 h. (**B**) % specific lysis was used to calculate killing frequency by dividing the number of target cells killed by the number of effector cells used for the 0.625:1 ratio.

To compare the killing potential of haNK cells to NK cells from healthy blood donors, we utilized MDA-MB-231 breast cancer cells as tumor targets, and NK cells from three healthy donors or haNK cells as effector cells. To determine the number of targets killed per effector cell, we used an ^111^In release assay at low E:T ratios in order for each effector cell to have access to excess target cells. As seen in Figure 6A, there were low levels of specific lysis mediated by NK cells from healthy donors at all E:T ratios. There were substantially greater levels of lysis observed from haNK cells at each ratio. Analysis of the killing frequency of NK cells from multiple donors demonstrated that on average 12 NK cells were required to kill one tumor target cell (Figure 6B). In contrast, only 4 haNK cells were required to kill one tumor target cell (*P* = 0.004), likely due to a greater probability for an individual effector cell to form contacts with target cells at lower E:T ratios.

**Figure 7:**
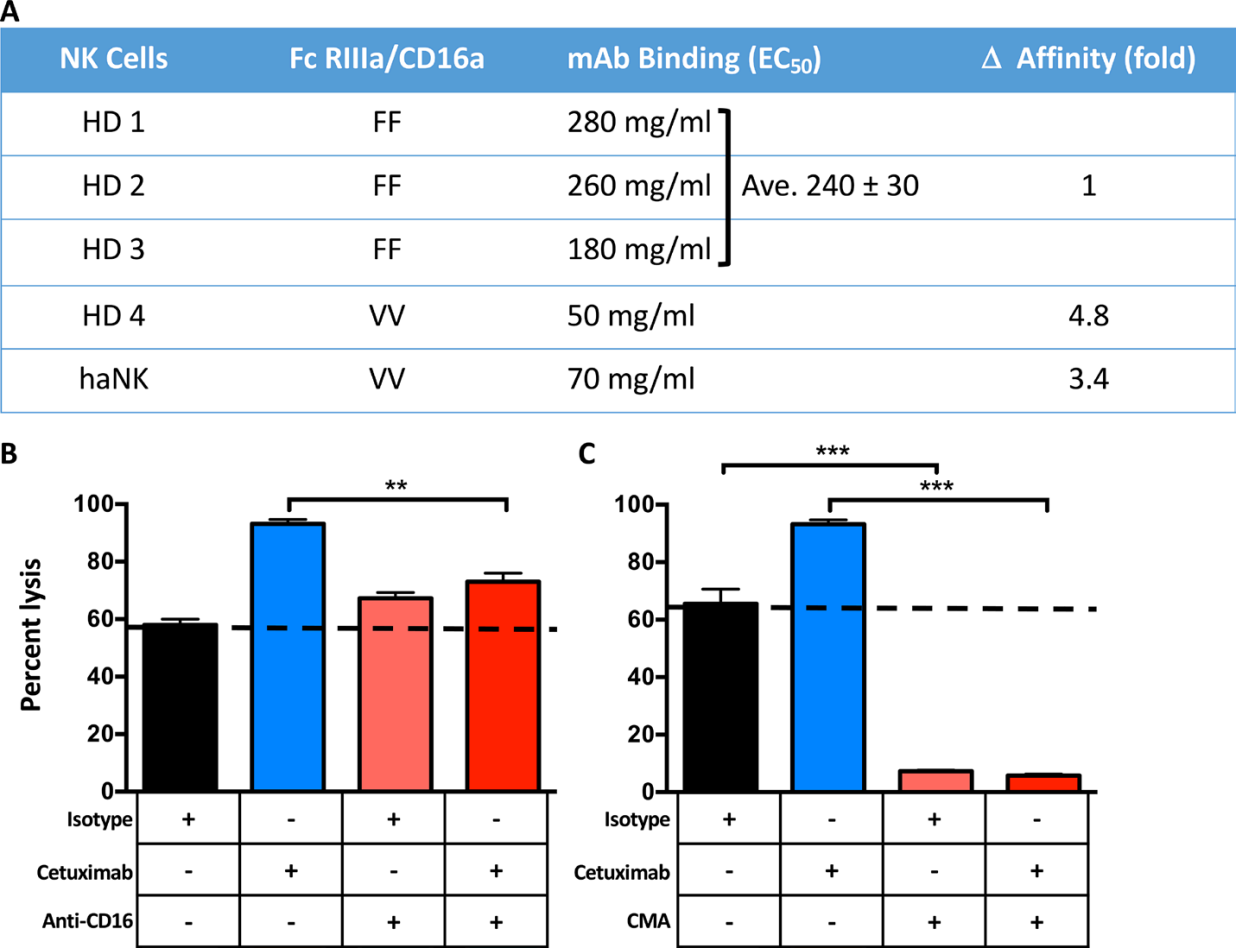
Comparative affinities of CD16 allotypes and factors influencing haNK-mediated lysis (**A**) Influence of CD16 *FCGR3A*-158V/F polymorphism on the concentration-effect relationship of cetuximab-dependent NK cell-mediated cytotoxicity. Purified NK cells from homozygous F/F, V/V healthy donors (HD) or haNK cells were incubated with varying concentrations of cetuximab for 30 min at 4°C followed by FITC-conjugated anti-CD16 3G8 MAb and then analyzed by flow cytometry. Percentages of inhibition of 3G8 binding were calculated as described in Methods, and the results are expressed as effective MAb concentration to achieve 50% inhibition of 3G8 MAb binding to NK cells (EC_50_). (**B** and **C**) The H460 human lung carcinoma cell line was used as a target in an 18-h ^111^In-release assay to evaluate if haNK ADCC mediated by cetuximab (10 μg/ml) could be blocked using anti-CD16 antibody (50 μg/ml) (B), or concanamycin A (CMA) (C). haNK cells were irradiated (10 Gy) and used at a 20:1 E:T ratio. Results shown are the mean (SEM) lysis of triplicate measurements in one of 3 repeat experiments. The dotted lines indicate endogenous haNK-mediated lysis. *T*-tests were employed to compare the treatments. ***P < 0.001, **P < 0.01.

Since haNK cells are engineered to express the CD16 high affinity FcγRIIIa receptor, one potential clinical utility would be the combined use of haNK cells with humanized or chimeric IgG1 anti-tumor MAbs. The binding of the anti-epidermal growth factor receptor (EGF-R) monoclonal antibody cetuximab (IgG1) to haNK cells was compared to the binding to NK cells of three healthy donors expressing homologous F/F alleles, and one donor expressing the high avidity V/V allele. Unfortunately, only one V/V donor was available since, as mentioned above, only approximately 10% of the population homozygously carries this allele [4, 14]. As seen in Figure 7A, the cetuximab EC_50_ binding and its calculated relative affinities for NK cells from the donor expressing the V/V allele and for haNK cells was approximately 4.8 and 3.4 times higher, respectively, than the binding to NK cells from the three donors expressing the F/F allele. Also note the variance in the EC_50_ binding to cetuximab seen with the NK cells from the three F/F donors.

ADCC of tumor cells is dependent in part on the interaction of CD16 of the effector cell with the Fc receptor of the anti-tumor IgG1 antibody. As seen in Figure 7B, the lytic activity of irradiated haNK cells on human lung carcinoma cells is enhanced by the addition of cetuximab. While the addition of a blocking anti-CD16 antibody did not reduce the lysis of the endogenous haNK lytic activity (isotype control Ab), it did reduce the level of cetuximab-mediated lysis (*P* < 0.01). In a separate series of studies, cetuximab again enhanced haNK endogenous lysis of lung cancer cells not seen with the addition of an isotype control antibody (Figure 7C). Concanamycin A (CMA) is known to inhibit perforin-mediated cell lysis. The addition of CMA greatly inhibited both the endogenous haNK lysis as well as the cetuximab-mediated ADCC (*P* < 0.001, Figure 7C). Experiments have also shown that haNK cells express less than 0.2% FasL. Taken together, these studies show that haNK tumor cell lysis alone, and via the ADCC mechanism, is mediated exclusively by perforin/granzyme.

**Figure 8:**
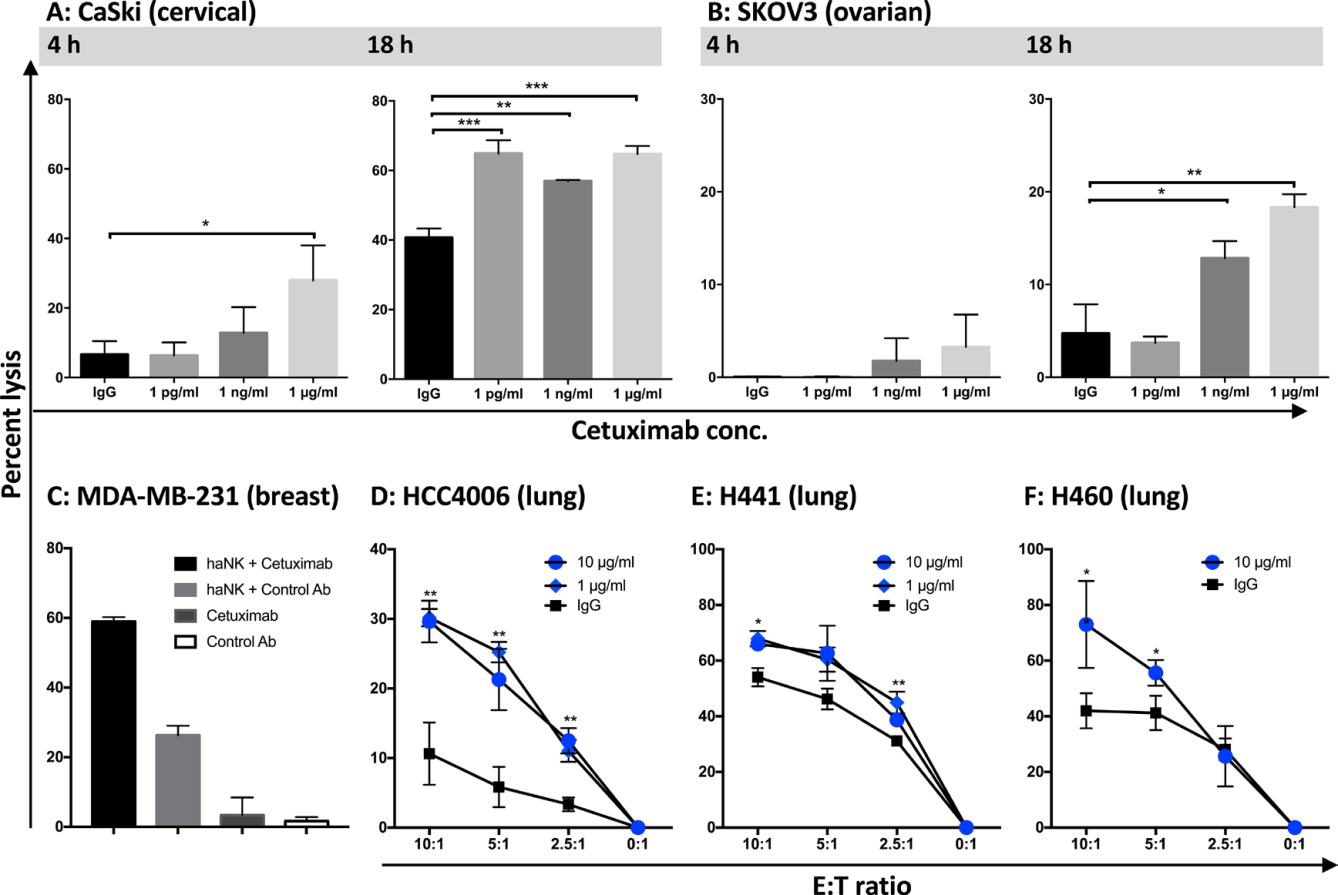
haNK ADCC mediated by cetuximab was evaluated with 4 h and 18 h 111In-release assays haNK cells (irradiated 10 Gy) were used as effector cells at different E:T ratios. Cetuximab was used at the specified concentrations. (**A**) CaSki: human cervical carcinoma (5:1 ratio); (**B**) SKOV3: human ovarian carcinoma (5:1 ratio). (**C**) MDA-MB-231 (human breast carcinoma) cells were used as a target at an E:T ratio of 7.5:1. Target cells were also incubated with cetuximab (1 μg/ml) or isotype control IgG1 antibody (1 μg/ml) alone, without effector cells. haNK ADCC mediated by cetuximab (black bar) and haNK killing (gray bar), cetuximab alone (dark gray bar) and control antibody alone (white bar) are shown. (**D**–**F**) 18-h lytic assays employing irradiated haNK cells with three different human lung carcinoma lines (HCC4006, H441, H460) at different E:T ratios. Results shown are the averages (SD) of triplicate measurements from one of at least three comparable repeat experiments. Multiple t-tests were used to compare each dose with IgG control at all E:T ratios. ***P < 0.001, **P < 0.01, *P < 0.05.

Since NK cells have previously been shown to be “serial killers,” i.e., one NK cell can lyse multiple target cells, both 4-h and 18-h ^111^In-release assays were carried out using irradiated haNK cells, plus or minus various concentrations of cetuximab. Employing the 4-h assay, both endogenous haNK lysis and cetuximab-mediated ADCC were very low using the cervical (CaSki) and ovarian (SKOV3) carcinoma cell lines as targets. Endogenous haNK lysis was improved using the 18-h assay, and the cetuximab-mediated ADCC further improved the levels of lysis in both cell lines in the 18-h assays (Figure 8A and 8B). Figure 8C provides further evidence that the addition of cetuximab to the haNK cell lysis is mediated by ADCC since little or no lysis was observed using cetuximab alone. Using the 18-h assay, the lysis of three additional lung carcinoma cell lines was observed with irradiated haNK cells, which was also improved with the addition of cetuximab and not isotype control antibody (Figure 8D–8F).

**Figure 9:**
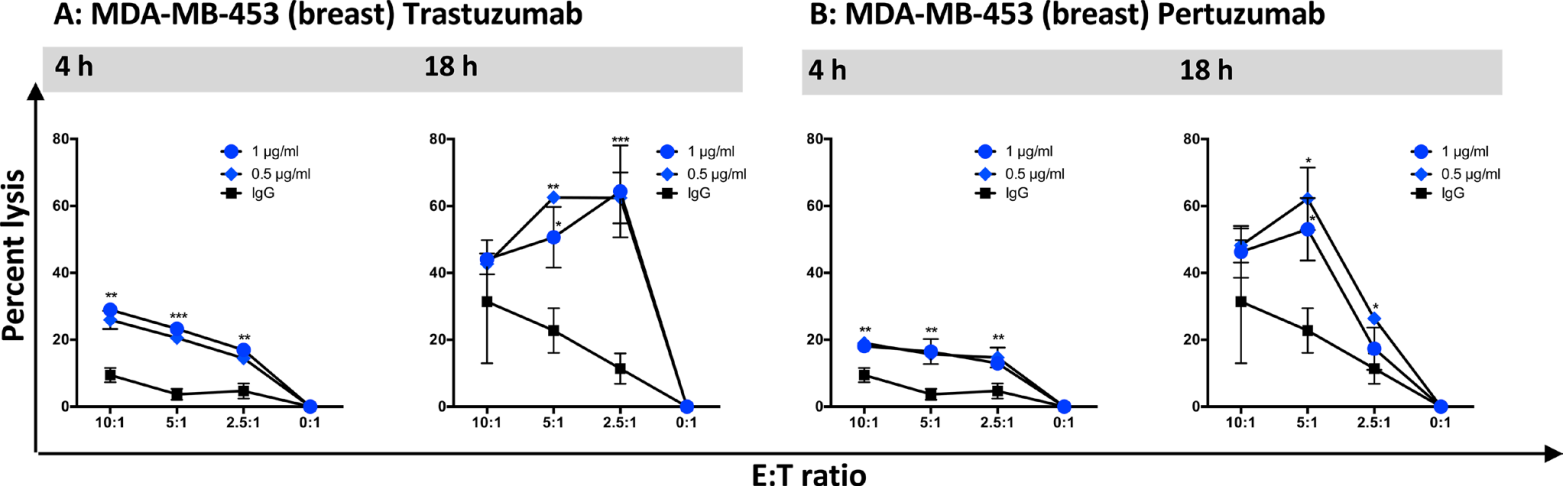
haNK cell ADCC mediated by trastuzumab and pertuzumab was evaluated with 4 h and 18 h 111In-release assays haNK cells (irradiated 10 Gy) were used as effector cells at different E:T ratios, and MAbs were used at the specified concentrations. MDA-MB-453: human breast carcinoma. Results shown are the averages (SD) of triplicate measurements from one of at least three comparable repeat experiments. Multiple *t*-tests were used to compare each dose with IgG control at all E:T ratios. ***P < 0.001, **P < 0.01, *P < 0.05.

We also evaluated the effects on haNK-mediated ADCC with two different IgG1 MAbs used in the therapy of breast cancer: trastuzumab (Herceptin) and pertuzumab (Perjeta). Using MDA-MB-453 human breast carcinoma cells as targets, both endogenous lytic activity of irradiated haNK cells and ADCC activity mediated by both monoclonal antibodies were enhanced in the 18-h vs. 4-h assay (Figure 9A and 9B). In the 18-h assays, the addition of either trastuzumab (Figure 9A) or pertuzumab (Figure 9B) greatly enhanced the lytic activity of haNK compared to the use of the isotype control antibody. Thus the use of three different antibodies directed against two diverse targets (EGF-R and human epidermal growth factor receptor 2 (HER2)) was shown to enhance the lytic activity of irradiated haNK cells.

## DISCUSSION

These studies describe the differences between haNK cells and the parental NK-92 cells in terms of the expression of the high affinity CD16 158V allele, and demonstrate high levels of granzyme and perforin in haNK cells. The lack of tumorigenicity of haNK cells in athymic mice and the kinetics of haNK cell growth in culture are also described. The kinetics of eliminating haNK viability by irradiation is described here for potential clinical utility. These results are similar to those observed with the parental NK-92 line [18, 19]. In preliminary experiments showing anti-tumor effects, 10^7 111^In-labeled irradiated haNK cells were injected i.v. in nu/nu mice bearing a human lung xenograft; approximately 2% ID/g was observed in tumor at 21 h, while the majority of cells accumulated in spleen and liver. haNK cells are shown to continue to produce IL-2 for 48 h post-irradiation; the levels of IL-2 produced by these cells are in the pg range and thus should not pose safety concerns. Nonetheless, dose escalating Phase I studies using irradiated haNK cells will involve careful monitoring of both serum cytokines and any changes in numerous peripheral immune cell subsets [20, 21] in addition to standard clinical analyses. The data presented here also show that the endogenous lytic ability of haNK cells is reduced immediately after irradiation (Figure 3), but returns to levels similar to non-irradiated haNK cells at 24 h (Figure 2F).

While most phenotypic markers such as CD56, CD16, NKG2D, Ki67, CD107a, and granzyme and perforin did not change with haNK irradiation, irradiated haNK cells did show enhanced expression of NKp44, PD-1, and PD-L1. NKp44 is a triggering receptor specifically expressed by activated NK cells. It is upregulated by IL-2, and mediates non-MHC-restricted tumor cell lysis [22]. PD-1 expression on NK cells is associated with activation and exhaustion. Previous studies have shown that subsets of NK cells expressing PD-1 display lower IFN-γ MFI and degranulation [23], which can be reverted by PD-1 blockade [23, 24]. In spite of the observed increase in PD-L1 expression after irradiation, there was no haNK fratricide of haNK cells induced by incubation with an IgG1 anti-PD-L1 antibody (unpublished data). In addition, we evaluated the expression of three activating and/or inhibitory KIR (CD158a, CD158d and CD158j), and found all to be below the detection limit. It should be noted that multiple phenotypic analyses have been carried out using different passages of haNK, different densities of haNK in culture, and haNK cells shipped under different conditions. There were minor differences observed in haNK phenotype as a consequence of the above situations; thus the data presented in Figure 4 should be considered representative and not absolute. Process development studies are underway to best standardize the phenotype of irradiated haNK cells.

Irradiated haNK cells (24 h post-irradiation) were also assayed for lytic activity in both 4-h and 18-h ^111^In-release assays; three of three human carcinoma cell lines showed greater levels of lysis in the 18-h assay (Figures 8 and 9). This result is compatible with the previously published results [16, 17] that NK cells are “serial killers.” We observed that on a per-cell basis, haNK cells had a 3-fold higher killing frequency than the average killing frequency of NK cells from three healthy donors (Figure 6). These data extend the observations of Bhat et al. where quality of effector cells was directly proportional to the killing frequency rate [16].

In this study, 21 of 22 different human tumor cell lines are shown (Figures 2, 3, 5, 6, 7, 8, 9, Table 1) to be lysed by haNK cells *in vitro*, including lung, head and neck, colon, breast, bladder, cervical, and ovarian carcinoma lines, and a chordoma cell line; a range of lysis was observed among lines. Some of the phenotypic characteristics of eight of these lines were analyzed. While there were trends in lysis correlating with increased MICA and PD-L1 expression, and decreased MHC class I expression, none were statistically significant (Table 1). Future studies are planned to analyze a range of human tumor cell lines by total RNA sequence and proteome to help identify which tumor cells, and eventually which tumors, are most susceptible to haNK-mediated lysis.

The insertion of the high affinity CD16 FcγRIIIa (158V) allele into haNK cells renders them capable of mediating ADCC. Humanized or chimeric MAb of the IgG1 isotype is the only isotype capable of mediating ADCC. It is of interest to note that four of the most widely used MAbs in cancer therapy are of this IgG1 isotype: cetuximab targeting the EGF-R in colorectal, head and neck, and lung carcinomas, rituximab targeting CD20 in lymphoma, and trastuzumab and pertuzumab targeting HER2/neu in breast carcinoma. As mentioned above, prior studies exist indicating potential ADCC involvement in the clinical activity of these antibodies in addition to the mechanism of receptor-ligand interactions, especially in the approximately 10% of patients who are homozygous for the V/V allele. It should be pointed out that prior studies [25–30] reported variable percentages in the frequency of the V/V allotype, ranging from approximately 8% to 30%. The differences reported could be due to the population analyzed; on the other hand, another study [31] reported that this variation is most likely due to differences in the methods employed, and analyses of samples from 42 donors in this study indicated a V/V allotype frequency of 14.3%. Studies in our own laboratory of peripheral blood mononuclear cells (PBMCs) of 70 donors showed a V/V allotype frequency of 8.8%. The adoptive transfer of irradiated haNK cells in combination with any of these monoclonals would add the V/V component to enhance ADCC-mediated tumor cell lysis. Obviously, well-controlled randomized studies will be required to analyze this possibility.

Ongoing preclinical studies and future clinical studies will undoubtedly use combinations of different cancer immunotherapy platforms, including anti-tumor MAbs, vaccines, checkpoint inhibitors, and immune modulators, to enhance effector functions or reduce or eliminate immunosuppressive entities. The studies described here are designed to provide a rationale for the use of irradiated haNK cells as a potential cancer immunotherapeutic, first as a single agent but, more importantly, in combination with either standard-of-care therapeutics or other immunotherapeutic modalities.

## MATERIALS AND METHODS

### Cell lines and cultures

The parental cell line NK-92 was originally established from a male 50-year-old patient with non-Hodgkin's lymphoma, whose bone marrow was infiltrated with large granular lymphocytes (LGL) [6]. NK-92 has consistently shown high antitumor cytotoxicity, and has been infused into approximately 50 cancer patients with clinical benefit and minimal side effects [4]. The NK-92 parental cell line is dependent on IL-2 for proliferation, and was therefore genetically modified to produce IL-2 in an autocrine loop, as well as to express a high affinity variant (158V) of the CD16 FcγRIII, in order to have a capacity for ADCC in addition to NK lysis [4, 15]. haNK cells were created by electroporation of NK-92 cells with a plasmid designed to co-express both CD16 (158V) and an internally retained form of IL-2 (ERIL-2) as a single bicistronic transcript. Expression of ERIL-2 haNK enables cell proliferation in the absence of IL-2 in the medium, and haNK cells can thus be continuously cultured in medium without IL-2 supplement. Stable expression of CD16 (158V) was maintained for over 3 months. haNK cells were provided through a Cooperative Research and Development Agreement (CRADA) between the NCI and NantBioScience (Culver City, CA). haNK cells were cultured in phenol-red free and gentamycin-free X-Vivo-10 medium (Lonza, Walkersville, MD) supplemented with 5% heat-inactivated human AB serum (Omega Scientific, Tarzana, CA) at a concentration of 5 × 10^5^/ml. Human tumor cell lines (H441, SKOV3, K562, H460, H1703, HCC4006, H520, SUM149, A549, ASPC-1, CaSki, SW480, Colo205, Caco2, MCF7, MUG-Chor1, HTB1, SW403, MDA-MB-231, MDA-MB-453, HN12, HN4, and HN30) were purchased from American Type Culture Collection (Manassas, VA). All cultures were free of mycoplasma and maintained in RPMI-1640 supplemented with 10% FCS, 100 U/ml penicillin, 100 μg/ml streptomycin, and 2 mM L-glutamine (Mediatech, Herndon, VA), except HN12, HN4, and HN30, which were cultured in DMEM (Mediatech). PBMCs from healthy donors were obtained from the NIH Clinical Center Blood Bank (NCT00001846).

### Immunofluorescence imaging

Live haNK cells were added to Cell-Tak (Corning, Bedford, MA) coated microscope slides at a concentration of 1 × 10^5^ cells per slide and incubated for 20 min. All incubations were performed at room temperature. Slides were tilted to remove excess cells and immediately fixed in 3% paraformaldehyde in PBS for 15 min. The slide stained with anti-CD16 (mouse) antibody was immediately blocked with PBS containing 3% bovine serum albumin (PBS-3% BSA). All other slides were permeabilized in 0.05% Triton-X100 in PBS for 15 min, then blocked in PBS-3% BSA for 30 min. Primary antibodies were added at the following dilutions in PBS-3% BSA for 2 h: anti-CD56 (mouse), anti-CD16 (mouse), anti-NKG2D (goat) were all obtained from Santa Cruz Biotech (Dallas, TX) and used at 1:500; anti-perforin (mouse, eBioscience, San Diego, CA) and anti-tubulin (mouse, Thermo Fisher Scientific, Rockford, IL) were both used at 1:250. Alexa Fluor 488, 568, and 647 conjugated secondary antibodies were obtained from Thermo Fisher Scientific and used at 1:1000 in PBS-3% BSA for 45 min. The Phalloiden 488 F-actin stain was added with the secondary antibodies at 5 μl per test. After secondary antibodies were removed, CellMask Deep Red Plasma membrane stain (Thermo Fisher Scientific) was added at 1:5000 in PBS for 15 min. Coverslips were mounted onto slides with ProLong gold antifade mountant with DAPI (Thermo Fisher Scientific) and allowed to dry overnight before imaging on a Zeiss LSM 710 NLO confocal microscopy system (Carl Zeiss Inc., Thornwood, NY).

### Evaluation of the tumorigenic potential of non-irradiated haNK cells

Male athymic nude mice (Taconic Biosciences, Hudson, NY) were injected s.c. with 10^6^ or 10^7^ tumor cells or non-irradiated haNK cells, and monitored daily for the presence of tumors at injection sites. The tumor cell lines were: MOLT-4 (acute lymphoblastic T-cell leukemia), Raji (Burkitt's lymphoma), Reh (acute lymphoblastic non-T/non-B cell leukemia), and Daudi (Burkitt's lymphoma). At study conclusion (day 63) the number of resultant viable tumors (≥ 50 mm^3^) was quantified for each tumor cell line and cell number injected.

### Evaluation of haNK cell viability and cytokine production

haNK cell proliferation after irradiation was evaluated by ^3^H-thymidine incorporation by pulsing the cells with ^3^H-thymidine for 4 h at 24 h, 48 h, and 72 h post-irradiation. The cells were then lysed for measurement of ^3^H incorporation. haNK cell viability was continuously monitored using dual-fluorescence for live/dead cells with acridine orange/propidium iodide (AO/PI) staining on a Cellometer Auto 2000 (Nexcelom, San Mateo, CA), according to the manufacturer's instructions. Release of IL-2 into culture supernatants was measured with an ELISA kit (AllCells, Alameda, CA).

### Antibodies and flow cytometric analysis

The cetuximab (Bristol-Myers Squibb, New York, NY), trastuzumab (Genentech, San Francisco, CA), pertuzumab (Genentech) and isotype (rituximab, Genentech) antibodies were purchased from the National Institutes of Health Pharmacy. Flow cytometry of haNK cells was performed on a BD LSRII flow cytometer (BD Biosciences, San Jose, CA) and analyzed in FlowJo 9.9 (TreeStar Inc., Ashland, OR). Flow cytometry of tumor cells was performed on a BD FACSVerse flow cytometer (BD Biosciences) and analyzed in BD FACSuite (BD Biosciences). Staining of haNK cells was performed with four panels, and the antibodies used were: anti-CD56-PerCP-Cy5.5, anti-CD16-APC-Cy7, anti-NKG2D-APC, anti-NKG2D-FITC, anti-CD107a-PE-Cy7, anti-GranzymeB-FITC, anti-perforin-APC, anti-NKp46-PE-Cy7, anti-NKp30-APC, anti-NKp44-PE, anti-2B4-FITC, anti-CD158a-PE, anti-CD158d-PE, anti-CD158j-APC, anti-PD-1-PE-Cy7, anti-PD-L1-APC, and anti-MICA-PE; all were obtained from BioLegend (San Diego, CA). Anti-CD56-PE, anti-CD16-PE-Cy7, CD11a-PE, Ki67-FITC, anti-HLA-ABC-FITC and anti-4-1BB-BV421 were obtained from BD Biosciences. In controlled experiments to detect CD16 on haNK cells, the anti-CD16 MAb was CD16-FITC (BD Biosciences) and the isotype control antibody was MIgG1k-FITC (BD Biosciences).

### NK lysis, ADCC, and blocking experiments

haNK cells were used as effectors in lysis assays non-irradiated, or irradiated (10 Gy) and assayed immediately post-irradiation or after 24 h, as specified for each experiment.

Lysis assays were performed for 4 h and 18 h, with either an ^111^In-release assay or calcein AM staining and analysis on a CeligoS instrument (Nexcelom, San Mateo, CA). For the ^111^In-release assays, target cells were labeled with 20 μCi of ^111^In-oxyquinoline (GE Healthcare, Chicago, IL) at 37°C for 20 min and used as targets at 3,000 cells/well in a 96-well round-bottom culture plate at various E:T ratios. For NK lysis experiments, haNK cells were added to the wells immediately after the target cells. For ADCC experiments, the targets were first incubated for 30 min with the MAbs before the haNKs were added, as previously described [32]. The plates were incubated for 4 h or 18 h at 37°C in a humidified atmosphere containing 5% CO_2_, then harvested and counted on a Wizard^2^ gamma counter (PerkinElmer, Shelton, CT). All samples were tested in triplicate and specific lysis was calculated from the average. Spontaneous release was determined by incubating targets with medium alone; complete lysis was determined by incubating targets with 0.05% Triton X-100 (Sigma-Aldrich, St. Louis, MO). Specific lysis was determined using the following equation:

Percent lysis = (experimental − spontaneous)/(complete − spontaneous) × 100.

For analysis on the CeligoS instrument, target cells were stained with calcein AM (10 μM) for 30 min at 37°C, washed and plated at 2-3,000 cells/well in a 96-well black plate with clear bottom according to the manufacturer's instructions. After the cells had adhered for 2 h, haNK cells were added, and the plate was incubated for 4 h or 18 h. Complete lysis was attained with 0.05% Triton X-100, and propidium iodide was added immediately before reading on the CeligoS instrument to evaluate cell death.

For killing frequency experiments, human NK cells were purified from three healthy blood donors by negative selection (Miltenyi Biotech, San Diego, CA) and rested at 37°C for 24 h. haNK cells were irradiated (10 Gy) and assayed after 24 h. NK or haNK cells were co-incubated with ^111^In-labeled MDA-MB-231 (breast cancer) target cells at low E:T ratios for 18 h.

For the blocking experiments, irradiated haNK cells were pre-incubated for 2 h with either anti-CD16 antibody (50 μg/ml, BD Biosciences) or concanamycin A (CMA; 200 nM, Sigma-Aldrich, St. Louis, MO) before being used in lysis assays with the H460 human lung carcinoma cell line as a target at a 20:1 E:T ratio. Both NK lysis and ADCC mediated by cetuximab (10 μg/ml) were performed in 18-h ^111^In release assays.

### CD16 (FcγRIIIa) genotyping

DNA was extracted from PBMCs of four healthy donors using a QIAamp DNA Blood Mini kit (Qiagen, Valencia, CA), and stored at −80°C until use. The polymorphism of CD16 at amino acid position 158 that is a valine vs. phenylalanine was determined using allele-specific droplet digital polymerase chain reaction (ddPCR) employing the TaqMan array for CD16 (rs396991; Life Technologies, Waltham, MA) [31, 33, 34]. A master reaction mix was prepared, and 1 mL of genotyping DNA was added. The PCR reaction was performed on a Bio-Rad T100 thermal cycler (Bio-Rad, Hercules, CA) for 40 cycles at 95°C for 10 min, 94°C for 30 sec, and 60°C for 1 min. The plate was read on a Bio-Rad QX200 droplet reader. Data were analyzed with Bio-Rad QuantaSoft v.1.5 software.

### Evaluation of CD16 binding affinity

NK cells from homozygous FF and VV healthy donors were isolated using the Human NK Cell Isolation (negative selection) Kit 130-092-657 (Miltenyi Biotech, San Diego, CA) following the manufacturer's instructions, resulting in > 90% purity. Purified NK cells or haNK cells were incubated with varying concentrations of cetuximab for 30 min at 4°C followed by FITC-conjugated anti-CD16 3G8 MAb, and then analyzed by flow cytometry. Percentages of inhibition of 3G8 binding were calculated and the results are expressed as the effective MAb concentration to achieve 50% inhibition of 3G8 MAb binding to NK cells (EC_50_) [35].

### Statistical analyses

Statistical analyses were performed in GraphPad Prism 6, using multiple *T*-tests, with a desired false discovery rate of 1.00%.
